# A systematic review of associations between functional connectivity, mood and cognition in patients with irritable bowel syndrome

**DOI:** 10.1007/s11682-026-01135-9

**Published:** 2026-04-07

**Authors:** Mahnaz Reisian, Catherine Toben, Liliana G. Ciobanu, Magdalene C. Jawahar, Sally Eldeghaidy, Scott R. Clark

**Affiliations:** 1 Discipline of Psychiatry, Adelaide University, Adelaide, SA Australia; 2 Basil Hetzel Institute, Woodville, Adelaide, SA Australia; 3 Division of Food, Nutrition and Dietetics, School of Biosciences, University of Nottingham, Nottingham, UK; 4 Sir Peter Mansfield Imaging Centre, School of Physics and Astronomy, University of Nottingham, Nottingham, UK

**Keywords:** Irritable bowel syndrome, fMRI, Depression, Anxiety, Cognition, Mood disorders, Neuroimaging, Functional brain connectivity

## Abstract

Irritable bowel syndrome (IBS) affects 9.3%–35.5% of the population, with women at greater risk. It is often comorbid with anxiety, depression, and cognitive dysfunction. Understanding the neural mechanisms underlying these comorbidities is crucial for identifying IBS pathology and developing targeted treatments. Functional magnetic resonance imaging (fMRI) offers valuable insights into brain connectivity and psychopathology. This review evaluates research on the link between functional connectivity, mood, and cognition in IBS. We systematically searched Embase, PubMed, and PsycINFO for studies published between 2010 and 2024, identifying 49 studies using both resting-state and task-based fMRI. Of these, 12 studies meeting inclusion criteria were reviewed. Across studies, IBS was associated with altered connectivity in the salience (SN), sensorimotor (SMN), default mode (DMN), and executive control (ECN) networks. Reported cognitive findings largely reflected executive and attentional control processes occurring in the context of pain anticipation, salience detection, and interoceptive awareness rather than domain-general cognitive impairment. Alterations in the SN, particularly involving the pregenual anterior cingulate cortex and anterior midcingulate cortex, were linked to increased visceral sensitivity and affective symptoms. Disrupted DMN connectivity was associated with altered self-referential processing and emotional regulation, while changes in the SMN and ECN suggested differences in sensory integration and top-down control. Notably, several studies showed that group differences in functional connectivity were reduced or no longer significant after accounting for anxiety and depression, suggesting that mood symptoms may play a mediating role in brain network alterations in IBS. While the literature is limited by small samples and sex imbalance, this review highlights a multi-network model of IBS that emphasizes emotional–cognitive–visceral interactions and points to important directions for future longitudinal research.

**Supplementary Information:**

The online version contains supplementary material available at 10.1007/s11682-026-01135-9.

## Highlights

This review examines both task-based and resting-state fMRI studies in IBS, focusing on brain alterations linked to mood and cognition.

Anxiety and depression are associated with abnormal activation in the cingulate cortex, insula, prefrontal cortex, and cerebellum during pain and non-painful visceral stimulation.

IBS patients show altered activity in the Executive Control Network (ECN) and Salience Network (SN) during cognitive tasks, including set-shifting and attention switching.

Resting-state fMRI reveals disrupted functional connectivity within and between the Default Mode Network (DMN), SN, and ECN in relation to mood symptoms.

IBS-related connectivity differences are often diminished after accounting for anxiety and depression, indicating that mood symptoms contribute meaningfully to observed network alterations.

**Supplementary Information:**

The online version contains supplementary material available at 10.1007/s11682-026-01135-9.

## Introduction

Irritable Bowel Syndrome (IBS) is a gastrointestinal condition that affects a large portion of the global population, with prevalence estimates varying from 9.3% to 35.5% (Quiroga-Castañeda et al., 2024). It should be noted that women are more commonly affected by IBS, with prevalence estimated to be between 14% and 24% (JohnBritto et al., 2024; Lee et al., 2001). The primary symptoms of IBS are irregular bowel activity and abdominal pain, including diarrhea (IBS-D), constipation (IBS-C), or mixed, altering diarrhea and constipation (IBS-M), each presenting unique challenges in diagnosis and treatment (Quiroga-Castañeda et al., 2024). Initially in the diagnostic process, clinicians typically conduct a series of medical investigations to rule out more serious illness, particularly where symptoms are accompanied by unexplained weight loss, significant change in bowel habit or where there is evidence of gastrointestinal bleeding (Lacy & Patel, 2017; Moayyedi et al., 2017). The Rome criteria are a set of symptom-based guidelines used to diagnose IBS that focus on recurrent abdominal pain related to bowel movements, along with changes in stool frequency or consistency over the past three months (Whitehead & Drossman, 2010). The severity of IBS is commonly assessed using the IBS Severity Scoring System (IBS-SSS), which rates symptoms like abdominal pain, bloating, stool dissatisfaction, and their effect on daily life, classifying them as mild, moderate, or severe (Wang et al., 2022).

Increased comorbidity of anxiety and depression with IBS symptoms compared with the general population, implicate the gut-brain axis. This complex neurohormonal pathway bidirectionally links the central nervous system (CNS) with the gastrointestinal tract, thereby enabling centralized control of peripheral gut functions. Studies indicate that people with more severe IBS symptoms face more psychological distress such as depression and anxiety, which can worsen their gastrointestinal problems (Gros et al., 2009; Rey & Talley, 2009). Studies also show that nearly 70% of individuals diagnosed with IBS under the Rome criteria have at least one psychological comorbidity, such as anxiety or depression. The severity of IBS symptoms tends to increase as psychological comorbidities multiply, with many individuals reporting increased level of depression, anxiety and cognitive difficulties (Goodoory et al., 2021), which can significantly impact daily functioning.(Gonsalkorale et al., 2004; Mayer et al., 2023).

### Mood and cognition

Mood and cognitive symptoms in IBS may be influenced by the bidirectional relationship between the gut and the brain, involving the autonomic and enteric nervous systems, the hypothalamic-pituitary-adrenal (HPA) axis, and the reciprocal engagement of neurotransmitter systems including serotonin, dopamine, and norepinephrine (Price et al., 2006; Wang & Wang, 2016). Furthermore, changes in gut microbiota can have central effects on neurotransmitter levels, specifically serotonin, gamma-aminobutyric acid (GABA), and dopamine, which together may contribute to increased anxiety and depression (Chen et al., 2022). Chronic stress, common in IBS due to unpredictable symptoms, leading to elevated cortisol levels, can further disrupt the HPA axis and increase risk of depression and anxiety (Sic et al., 2024). Additionally, individuals with IBS have elevated levels of inflammatory markers such as interleukin-6 (IL-6) and tumor necrosis factor-alpha (TNF-α), which are also associated with depressive syndromes (Labanski et al., 2020; Ng et al., 2018). These cytokines can pass the blood-brain barrier, causing neuroinflammation, mood disturbances, and negatively impacting memory and executive functions (Kennedy et al., 2012; Lam et al., 2019). Understanding how specific components of the gut-brain axis and stress response, contribute to depression, anxiety, and cognitive performance is essential for developing effective treatments for IBS.

### Brain connectivity

Complex neural circuits or networks communicate and coordinate to support various functions such as sensory processing, pain perception, emotional regulation, and executive function (Gu et al., 2018; Palomero-Gallagher & Amunts, 2022). These neural networks are interconnected through brain regions that specialize in specific functions, and their coordinated activity is essential for healthy cognitive and emotional processes (Herbet & Duffau, 2020). Among the major networks identified are the Default Mode Network (DMN), Salience Network (SN), Executive Control Network (ECN), Sensorimotor Network (SMN), Visual Network (VN), Auditory Network (AN), as well as the Dorsal and Ventral Attention Networks (DAN and VAN). The DMN, SN, and ECN, in particular, play central roles in cognitive and emotional regulation (Domagalik-Pittner 2017; Li et al. 2023a, b). One of the key functional properties of neural networks is their connectivity, which can be assessed using neuroimaging techniques such as functional Magnetic Resonance Imaging (fMRI)(Bagherzadeh et al., 2022). fMRI measures change in blood flow and oxygenation associated with neural activity, providing a proxy measure of brain function and connectivity by identifying coordinated activity across different brain regions (Soares et al., 2016). There are two main methods of fMRI used to study brain connectivity: resting-state fMRI (rs-fMRI) and task-based fMRI. Resting-state fMRI measures spontaneous brain activity when the participant is not performing any specific task, offering insights into the intrinsic connectivity of functionally related brain regions, even without external stimuli (Smitha et al., 2017). On the other hand, task-based fMRI assesses brain activity during specific tasks or stimuli, allowing researchers to examine how brain regions communicate during task-directed responses (Zhang et al., 2016).

Understanding these functional connectivity patterns is crucial for explaining the neural mechanisms underlying the complex interaction between gastrointestinal and psychological symptoms in IBS. It has been shown that IBS patients exhibit altered connectivity in brain areas associated with pain processing, such as the anterior cingulate cortex (ACC), insula, and prefrontal cortex, which are also linked to cognitive control and emotional regulation (Nisticò et al., 2022; Weng et al., 2017). Several studies have described the association between mood disorders, cognitive function, and altered brain connectivity in IBS (Aizawa et al., 2012; Li et al., 2021). Specifically, changes in some brain areas like the pregenual anterior cingulate cortex (pACC) have been linked to mood regulation and cognitive dysfunction in IBS patients (Elsenbruch et al., 2010; Yang et al., 2023). While previous research has identified complex patterns of interactions between mood and cognition in IBS, to our knowledge, no studies have specifically integrated findings regarding mood, cognitive function, and functional brain connectivity in IBS. This systematic review aims to synthesize existing fMRI studies to identify brain regions with altered activity and connectivity associated with depression, anxiety, and cognitive function in individuals with IBS, and to map these regions to their corresponding functional neural networks based on established associations.

## Method

We followed the Preferred Reporting Items for Systematic Reviews and Meta-analyses (PRISMA) guidelines to conduct a review to investigating the relationship between neural connectivity, cognition and mood symptoms in individuals IBS (Page et al., 2021).

### Search strategy

A bibliographic search was conducted in databases PubMed, EMBASE, and PsycINFO in July 2024. We used specific keyword combinations, including key terms: (IBS), (irritable bowel syndrome), (brain mapping), (brain network), (brain function network), (attention network), (cognitive control network), (resting state network), (neural network), (brain connectivity), (fMRI), (functional MRI), (functional Magnetic Resonance Imaging), (depression), (depressive symptoms), (mood disorder), (anxiety), (stress disorders), (anxiety disorders), (anxiety and stress scale), (anxiousness), (cognition), (awareness), (cognitive reflection), (processing speed), (executive function), (attention), (cognitive performance), (cognitive processes), (cognitive ability), (memory), (executive control).

### Inclusion and exclusion criteria

We included peer-reviewed articles written in English, involving adult human subjects diagnosed with IBS who met the Rome criteria, compared to healthy controls without organic disease or psychiatric disorders. Only studies that used fMRI methods and examined the association of anxiety, depression, or cognition with brain connectivity were included. Behavioral outcomes had to be assessed using validated tools. For depression, studies used scales such as the Hamilton Depression Rating Scale (HAMD) and the Self-Rating Depression Scale (SDS). Anxiety was assessed with tools such as the Hamilton Anxiety Scale (HAMA) and the State-Trait Anxiety Inventory (STAI). The studies were published between 2010 and 2024, a period when resting-state fMRI became more commonly used due to improvements in data quality and analysis methods (Van Dijk et al., 2010).

We excluded review articles, conference papers, structural MRI studies (i.e., studies without fMRI), and studies that did not include healthy controls with behavioral measurements. To minimize pharmacological influences on brain activity, we also excluded studies that did not consider medication use as an exclusion criterion.

### Data extraction

Data were extracted using a standardized review table, including sample characteristics, study quality metrics, analyses techniques, and main findings. All articles were searched by author MR for inclusion/exclusion criteria under the supervision of LC, CT, and SC. MR and SC dual extracted data and inconsistencies were discussed with LC and CT.

### Risk of bias assessment

We evaluated the quality of the included studies using a process developed by Nichols et al. (2017) for applying the Joanna Briggs Institute (JBI) Critical Appraisal Tools (Nichols et al., 2017). Six categories of assessment included: research question clarity, recruitment methods, eligibility criteria, participant characteristics, imaging techniques, control group description. Each category was rated as having low risk, medium risk, or high risk of bias.

### Identifying brain networks and functional connectivity in IBS

To categorize findings, we first grouped studies based on imaging modality: task-based fMRI and resting-state fMRI. Resting-state studies were further subdivided by analytical approach, either using region-of-interest (ROI) analysis or whole-brain connectivity methods. Additionally, we classified studies by whether clinical/behavioral and imaging data were analyzed within integrated neuroimaging software (e.g., SPM, using clinical/behavioral scores as a covariate of interest) or using external correlation analysis (where brain activation in predefined ROIs were extract for each subject and corelated with clinical/behavioral scores) using statistical software (e.g. SPSS). Given the complexity and overlap among brain networks, we focused our synthesis on networks with well-established links in cognition and emotion that are particularly relevant to IBS. These include DMN, SN, SMN and ECN (Fox & King, 2018). Some regions not typically considered core nodes of these networks, such as the cerebellum and brainstem, were assigned based on their most consistent functional connections to cortical networks, without implying they are central members of those networks (Guell & Schmahmann, 2020; Singh et al., 2022).

## Results

### Study characteristics

**Fig. 1:**
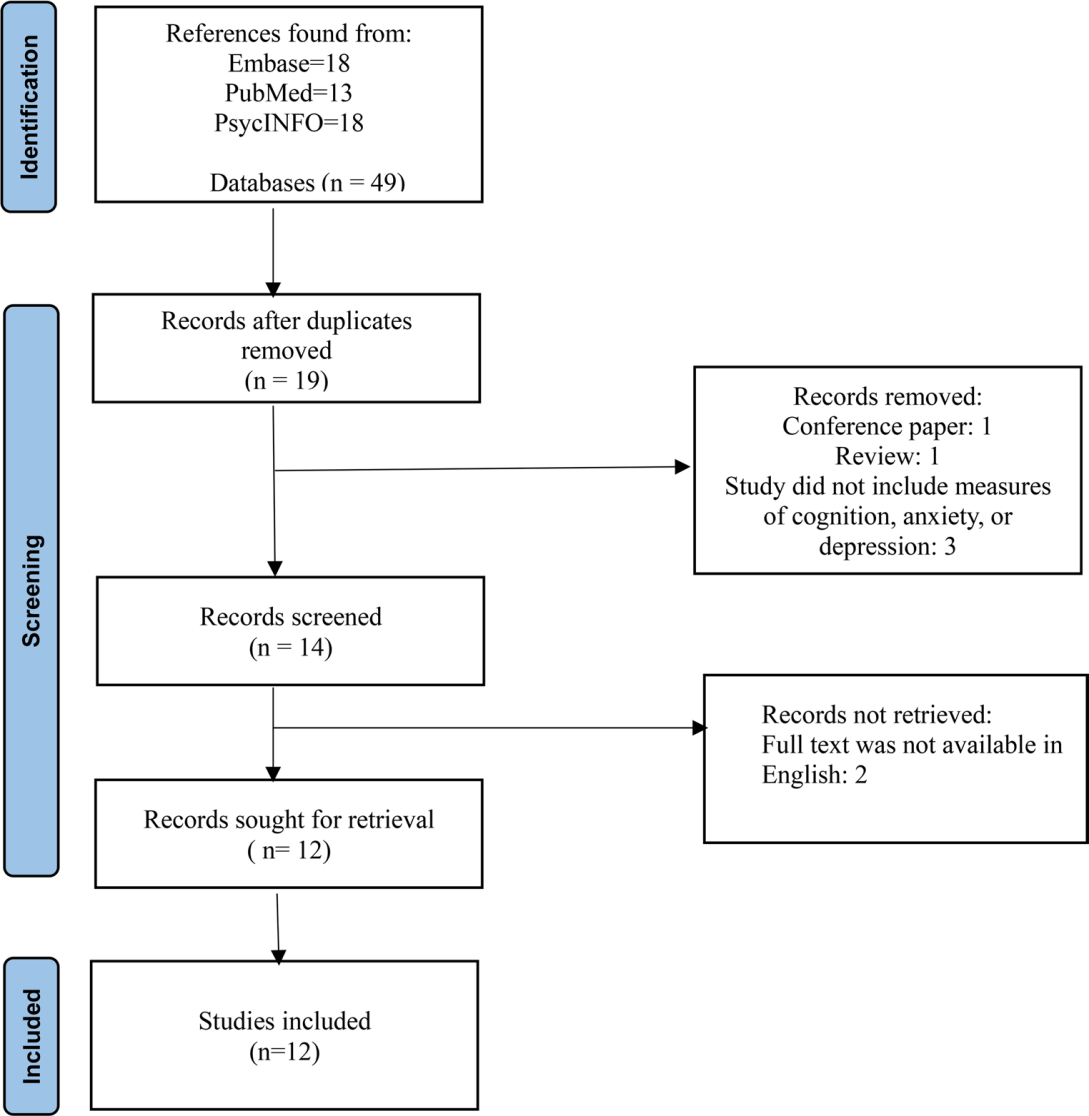
Prisma flow diagram

A total of 49 studies were initially identified. After removing 30 duplicates, the remaining studies underwent further assessment of eligibility criteria. We excluded 1 review paper, 1 conference paper, and 3 papers that did not match the research topic. From the remaining 14 studies, 2 were excluded due to the unavailability of full texts in English. Finally, 12 case-controlled fMRI studies were included in this systematic review. (PRISMA screening process described in Fig. 1)

Table 1 summarizes the characteristics of the 12 included studies, which included a total of 784 participants, with sample sizes ranging from 21 to 65 participants per study. Among these, 4 were task-based fMRI studies(Aizawa et al., 2012; Elsenbruch et al., 2010; Hubbard et al., 2015; Rosenberger et al., 2013) and 8 were resting-state fMRI studies(Chen et al., 2021; Icenhour et al., 2017; Ke et al., 2015; Li et al., 2021; Qi et al. 2016a, b, c, d). Most studies showed that the level of depression and anxiety was higher in IBS group than control group (Chen et al., 2021; Elsenbruch et al., 2010; Icenhour et al., 2017; Ke et al., 2015; Yang et al., 2023). Four studies focused exclusively on females with IBS (Elsenbruch et al., 2010; Hubbard et al., 2015; Icenhour et al., 2017; Rosenberger et al., 2013), while in other studies, the majority of participants were male (Li et al., 2021; Qi et al. 2016a, b, c, d; Yang et al., 2023).

**Table 1:** Task- based fMRI and rs-fMRI study characteristics

Author	Sample Characteristics	IBS/HC(*n*) Female sex ratio (%)	IBS symptoms duration	Mean Age(years) IBS/HC	Clinical measures	fMRI Method	Type of analysis
Task-based fMRI							
Elsenbruch, S, 2010 (Elsenbruch et al., 2010)	15/12	100%	over 10 years for 40% (6 patients) and 2–5 years for 53.3% (8 patients).	42.4/31.4	HADS	Stimulus-related fMRI/rectal distension	(ROI)-based analysis on IC, PFC, amygdala, thalamus, Subregions of the cingulate cortex (ACC, MCC, PCC) Exploratory and whole-Brain regression analysis
Aizawa, E, 2012 (Aizawa et al., 2012)	30/30	50%/50%	8.7 ± 1.0 years	21.7/21.4	POMS depression-dejection/POMS tension-anxiety	Task-related fMRI/Wisconsin Card Sorting Test	(ROI)-based analysis on DLPFC, ACC, OFC, pre-SMA, Caudate nucleus Using clinical/behavioral scores as a covariate of interest
Rosenberger, C, 2013(Rosenberger et al., 2013)	15/12	100%	Didn’t report	42.4/31.4	HADS	Stimulus-related fMRI/rectal distension	(ROI)-based analysis on Cerebellar lobules Using clinical/behavioral scores as a covariate of interest
Hubbard, C, 2015 (Hubbard et al., 2015)	15/14	100%	10.4 years	31.9/32.0	HADS, VSI, PSQ, PHQ15, STAI	Task-related fMRI/attention network test	(ROI)-based analysis on aINS, pINS, aMCC, ACC, mPFC, SFG, IFG Using clinical/behavioral scores as a covariate of interest
Rs-fMRI							
Ke, J, 2015 (Ke et al., 2015)	31/32	19%/21%	32.7 ± 23.6 months	27.5/29.2	SDS/SAS	Resting state-fMRI	ReHo analyses Using clinical/behavioral scores as a covariate of interest
Qi, R, 2016 (Qi et al. , 2016 a, b, c, d)	31/32	19%/21%	32.67 ± 23.56 months	29.23/27.47	SDS/SAS Mini-Mental State	Resting state-fMRI	Whole-Brain Resting-State Functional Using clinical/behavioral scores as a covariate of interest
Qi, R, 2015 (Rongfeng Qi, Chang Liu, Jun Ke et al., 2016)	30/31	20%/22%	20.40 ± 18.75 months	28.93/26.87	SDS/SAS Mini-Mental State Examination, Montreal Cognitive Assessment used to exclude participants with cognitive impairment	Resting state-fMRI	ALFF analysis seed-based inter-regional FC analysis Using clinical/behavioral scores as a covariate of interest
Qi, R, 2015 (Rongfeng Qi, Jun Ke et al., 2016)	31/32	19%/21%	Didn’t report	29.23/27.47	SDS/SAS Mini-Mental State Examination, Montreal Cognitive Assessment used to exclude participants with cognitive impairment.	Resting state-fMRI	network-based functional connectivity analysis (topological configuration analyses of DMN) Using clinical/behavioral scores as a covariate of interest
Qi, R, 2016 (Rongfeng Qi, Chang Liu, Yifei Weng et al., 2016)	65/67	25%/24%	Didn’t report	34.0/31.21	SDS/SAS	Resting state-fMRI	VMHC (Voxel-Mirrored Homotopic Connectivity) Analysis Offline correlation analysis
Icenhour, A, 2017 (Icenhour et al., 2017)	21 Hypersensitive, 20 Normosensitive/20	100%	Didn’t report	36.48/32.25	HADS, IBSS, VSI	Resting state-fMRI	Independent component analysis, ROI on amygdala, thalamus, aMCC, insula, ACC Offline correlation analysis
Li, J, 2021(Li et al., 2021)	49/36	41%/28%	depressed IBS: 20.64 ± 9.01 months Non-depressed IBS: 18.57 ± 4.00 months	34.32/31.67	HAMD-17	Resting state-fMRI	graph theory-based analysis using degree centrality (DC) Offline correlation analysis
Chen, X, 2021 (Chen et al., 2021)	36/36	55%/72%	19.11 ± 6.98 months	34.36/31.67	HAMD-17/HAMA-14, GSRS, VAS	Resting state-fMRI	ALFF (Amplitude of Low-Frequency Fluctuations) Analysis ReHo (Regional Homogeneity) Analysis FC (Functional Connectivity) Analysis (ROI) on Right lingual gyrus, SFG, right postcentral gyrus, right IPL, right SMA, right cerebellar posterior lobe, right midbrain, Left precuneus, right supramarginal gyrus Offline correlation analysis

*HADS* Hospital Anxiety and Depression Scale, *POMS* Profile of Mood States, *SAS* Self-Rating Anxiety Scale, *SDS* Self-Rating Depression Scale, *PSQ* Perceived Stress Questionnaire, *PHQ15* Patient Health Questionnaire, *HAMD* Hamilton depressive rating scale, *STAI* State-Trait Anxiety Inventory, *HAMA* Hamilton anxiety scale, *GSRS* Gastrointestinal Symptom Rating Scale, *VAS* Visual Analog Scale, *VSI* Visceral Sensitivity Index, *QOL* Quality of Life, *aMCC* anterior midcingulate cortex, *pACC* Pregenual anterior cingulate cortex, *PFC* prefrontal cortex, *IC* Insular cortex, *DLPFC* Dorsolateral Prefrontal Cortex, *OFC* Orbitofrontal Cortex, *pre-SMA* Pre-Supplementary Motor Area, *SFG* Superior Frontal Gyrus, *IFG* Inferior Frontal Gyrus, *IPL* Inferior Parietal Lobule

Most studies reported IBS symptoms for more than one year, with durations ranging from 8.7 ± 1.0 years to 32.7 ± 23.6 months. Additionally, Participants in the IBS group had an average age of 32.75 years.

Seven studies conducted correlations between imaging and clinical data, integrated within the imaging analysis software. In contrast, five studies extracted imaging data and performed correlation with clinical measures using external statistical tools.

### Study quality review

A quality assessment of the 12 included studies revealed that all studies demonstrated a low risk of bias, indicating robust methodology and reporting (see supplementary table 1) (Aizawa et al., 2012; Chen et al., 2021; Elsenbruch et al., 2010; Hubbard et al., 2015; Icenhour et al., 2017; Ke et al., 2015; Li et al., 2021; Qi et al. 2016a, b, c, d; Rosenberger et al., 2013).

### Neuroimaging

Overall, findings suggest that differences in brain activity and connectivity, specifically within and between the DMN, SN, SMN, and ECN, in patients with IBS is closely linked to symptoms of anxiety, depression and cognitive function (see Tables 2 and 3).

**Table 2:** Summary of findings and conclusions from reviewed studies

Author	Findings	Conclusions
Elsenbruch, S, 2010 (Elsenbruch et al., 2010)	• Anxiety scores: Linked to ↑ activation in aMCC and pACC during pain processing (*p* < 0.05). • Depression scores: Associated with ↑ pain-related activity in left PFC and cerebellum (*p* < 0.05). • ↑ Activity in left aIC and PFC in IBS (*p* = 0.026, 0.037) was non-significant after adjusting.	• People with IBS commonly report pain, often linked to depression and anxiety. • ↑Activity in pACC (linked to unpleasant experiences/autonomic responses) and mACC (involved in fear, negative anticipation, and avoidance) contribute to visceral hypervigilance. • In IBS, depression is associated with ↑ activity in inferior and medial PFC, regions involved in anticipating and processing unpleasant stimuli.
Aizawa, E, 2012 (Aizawa et al., 2012)	• No significant group differences in anxiety (*p* = 0.21) or depression (*p* = 0.12). • IBS patients made more set maintenance and persevering errors (*p* < 0.05). • ↓ Right DLPFC and hippocampal activity, ↑ left posterior insula activity during error feedback in set-shifting (*p* < 0.01). • IBS showed impaired DLPFC → pre-SMA modulation and ↑ intrinsic pre-SMA–caudate connectivity (*p* = 0.001).	• ↑ Errors in memory, attention, and inhibitory control in IBS are linked to altered DLPFC, insula, and hippocampal activity. • These changes may relate to anxiety and catastrophe. • ↑ Right DLPFC activity is associated with anticipating/extinguishing visceral pain and modulating nociceptive perception.
Rosenberger, C, 2013 (Rosenberger et al., 2013)	• Anxiety scores correlated with non-painful activation of Crus II near VIIb (*p* = 0.01), no significant effects for painful distension. • Depression scores were linked to activation in right Crus II, VIIIb, and Crus I during non-painful distension. • Painful distension activated vermal lobule V, intermediate cerebellum, and smaller regions in lobules I–IV, VI, Crus I, VIIb, VIIIa (right), and Crus II (left) (all *p* < 0.01, uncorrected).	• Visceral stimuli activate cerebellar sensorimotor areas and PFC-linked regions, potentially impacting emotional processing. • Anxiety and depression may modulate cerebellar, subcortical, and cortical responses to these signals.
Hubbard, C,^2015^ (Hubbard et al., 2015)	• Alerting: ↑ aMCC, aINS, pINS activation • Orienting: ↓ IFJ and SMA activation • VSI and PCS correlated with aMCC, aINS, pINS, DMPFC, and thalamus activity • Fear of uncertainty linked to thalamus and DMPFC activity • aMCC activation during alerting: • A negative correlation with GI symptom duration (*r* = −0.69, *p* = 0.007) • Positively with symptom severity (*r* = 0.45, *p* = 0.09)	• IBS patients had faster reaction times in alerting and orienting, with no differences in executive control. • ↑ Activation in left aMCC, bilateral aINS, and right pINS during alerting reflects heightened salience network engagement tied to pain monitoring and cognitive control; this correlated with symptom severity, VSI, and PCS. • Right pINS activity was linked to harm avoidance, suggesting ↑ visceral vigilance. • ↓ Activity in left SMA, IFJ, and thalamus, with ↑ DMPFC activation, indicates impaired error processing and attentional control related to fear of uncertainty. • These attention network abnormalities suggest dysregulated executive function, contributing to anxiety, hypervigilance, and visceral hypersensitivity in IBS.
Ke, J, 2015 (Ke et al., 2015)	IBS patients showed: • ↑ ReHo in bilateral postcentral gyri, calcarine, vermis, right thalamus, left superior parietal lobule • ↓ ReHo in aMCC/pACC, sACC/vmPFC, dlPFC, vlPFC, right caudate, angular gyrus • Controlling for SAS and SDS had no effect on results.	IBS patients exhibit disrupted local synchronization of brain activity in regions linked to visceral processing, emotional arousal, and cognitive regulation.
Qi, R, 2016 (Qi et al. 2016 a, b, c, d)	• IBS patients showed ↑ resting-state connectivity of left and right amygdala with: Insula, midbrain, precentral gyri, SMA • Anxiety, depression, age, sex, and education had no effect on these relationships.	IBS patients show ↑ resting-state connectivity between the amygdala and corticolimbic regions, likely linked to heightened emotional arousal and visceral processing, independent of depression or anxiety.
Qi, R, 2015 (Rongfeng Qi, Chang Liu, Jun Ke, et al., 2016)	IBS patients showed: • ↓ ALFF in MPFC, PCC, IPC, MFC, ACC • ↑ ALFF in posterior insula, cuneus • ↓ Positive FC: MPFC–ORBsup, vACC–PCC • ↓ Negative FC: MPFC–posterior insula • ↑ Negative FC: MPFC–cuneus • These results were unaffected by anxiety, depression, age, sex, or education.	• ↓ ALFF in DMN (MPFC, PCC, IPC) • ↑ ALFF in insula and cuneus • ↑ Negative FC between MPFC and cuneus, indicating overactivity in pain processing and compensatory MPFC response • Higher depression and anxiety were linked to dysregulation in the ACC, with altered activity in dACC and vACC, regions involved in error detection, working memory, attention, and emotional regulation.
Qi, R, 2015 (Rongfeng Qi, Jun Ke, et al., 2016)	IBS patients showed: • ↓ FC within DMN, including ACC–precuneus, ORBsupmed–precuneus, middle temporal gyrus–precuneus (*p* < 0.005) • DMN exhibited small-world topology with stronger local than global connections • ↓ Average global connectivity (F = 6.99, *p* = 0.01) Anxiety and depression covariates: • Reduced FC differences between left ORBsupmed and left precuneus • Partially corrected global connectivity	• IBS patients showed reduced FC within the DMN, particularly between: Precuneus and ACC, Some prefrontal regions • Disruption was more prominent in long-range connectivity, especially between anterior frontal and posterior regions (e.g., bilateral precuneus), leading to fewer inter-regional connections within the network.
Qi, R, 2016 (Rongfeng Qi, Chang Liu, Yifei Weng, et al., 2016)Qi, R., Liu, C., Weng, Y., Xu, Q., Chen, L., Wang, F., Zhang, L. J., & Lu, G. M. (2016). Disturbed interhemispheric functional connectivity rather than structural connectivity in irritable bowel syndrome. Frontiers in molecular neuroscience, 9, 141.	IBS patients showed: • ↓ VMHC in bilateral vACC and IPL • ↑ VMHC in thalami, cuneus, PCC, lingual gyri, occipital/cerebellar lobes • Including anxiety, depression, age, sex, and education as covariates corrected the vACC VMHC difference.	IBS is linked to functional disorganization rather than structural changes, though structural connectivity findings show variability. VMHC abnormalities are mainly seen in the cortex-thalamus circuit: • ↑ VMHC in thalamus, cuneus, PCC, lingual gyrus, occipital/cerebellar lobes • ↓ VMHC in vACC and IPL Enhanced interhemispheric FC between the thalami and ↑ VMHC in the homeostatic afferent network suggest heightened visceral processing. This network connects sensory input (brainstem), thalamus, insula, and aMCC, mediating affective and motor responses. • ↑ VMHC in cuneus and PCC supports these findings • ↓ VMHC in vACC suggests affective dysregulation linked to depression and anxiety
Icenhour, A,^2017^ (Icenhour et al., 2017)	Hypersensitive IBS patients showed altered brain connectivity: • ↑ FC in right amygdala and mid-insula • ↓ FC in left dorsal anterior insula • These changes were linked to IBS symptom severity, urgency, and pressure tolerance, but no significant correlations with anxiety or depression were found.	• In the DMN: ↓ FC between amygdala and dorsal anterior insula in normosensitive IBS patients suggest corticolimbic inhibition, downregulating attentional and emotional processes. • In the SN: ↑ Connectivity between pACC and thalamus may reflect enhanced attentional and emotional resources in visceral afferent signalling during resting conditions in hypersensitive patients. • In the SMN: ↑ Activation of the posterior insula likely indicates amplified visceral signal processing in patients with visceral hypersensitivity.
Li, J, 2021 (Li et al., 2021)	DEP-IBS patients showed: • ↓ DC in left insula • ↑ DC in left precentral gyrus (compared to HC and nDEP-IBS) • ↓ DC in left medial superior frontal gyrus in both IBS groups • Female DEP-IBS and nDEP-IBS patients had ↓ DC in left precentral gyrus (DEP-IBS: *P* = 0.011; nDEP-IBS: *P* = 0.040) • DC in left insula negatively correlated with HAMD scores (*r* = − 0.524, *P* = 0.009) FC analysis: • ↑ FC between left insula and right inferior occipital gyrus • ↓ FC with right SMA and precentral gyrus in DEP-IBS • ↑ FC with left inferior parietal lobule • ↓ FC with ACC • FC between left insula and right SMA negatively correlated with HAMD scores (*r* = − 0.438, *p* = 0.037)	• DEP-IBS patients show abnormal FC in fronto-limbic and sensorimotor networks, particularly involving the insula and SMA. These changes may create a feedback loop between negative emotions and gastrointestinal symptoms. • FC changes in the left insula are linked to chronic visceral pain, involving areas like the ACC, somatosensory cortex, and posterior parietal cortex. • Abnormal FC between insula and SMA is associated with depressive symptoms. • Lower DC in the left precentral gyrus (especially in females) reflects depression and visceral pain perception. • ↓ DC in the left mPFC suggests impaired pain suppression.
Chen, X, 2021 (Chen et al., 2021)	IBS-D patients showed altered brain activity and connectivity compared to healthy controls (HC): ALFF: • Increased: Right cerebellum, lingual gyrus, postcentral gyrus, superior & middle frontal gyri. • Decreased: Right inferior parietal lobule, striatum, ACC, insula, hippocampus, thalamus, midbrain, left precuneus. ReHo: • Increased: Bilateral lingual gyrus, superior frontal gyrus, right middle frontal gyrus, postcentral gyrus. • Decreased: Left orbital inferior frontal gyrus, right SMA. • Connectivity: Enhanced connectivity between left precuneus & bilateral orbitofrontal cortex. • Correlation: ALFF in the right midbrain correlated with depression (HAMD: *r* = 0.506, *p* = 0.003), anxiety (HAMA: *r* = 0.349, *p* = 0.047), and gastrointestinal symptoms (GSRS: *r* = 0.501, *p* = 0.003), with GI symptoms mediating these relationships.	• IBS-D patients show disrupted spontaneous activity and functional connectivity (FC) in key areas related to pain regulation and emotional processing, particularly in the prefrontal-limbic-midbrain circuit and somatosensory networks. • Decreased ALFF in limbic regions is involved in pain regulation and emotional processing. Areas affected: ACC, insula, hippocampus, basal ganglia, thalamus. • Linked to chronic visceral hypersensitivity, emotional, and cognitive impairments. • Decreased ALFF in the right midbrain: Suggests dysfunction in the descending pain inhibitory pathway. May contribute to visceral hypersensitivity, abdominal pain, and depression. • This highlights the interplay between sensory, emotional, and cognitive processes in IBS-D.

aMCC: anterior midcingulate cortex, pACC: pregenual anterior cingulate cortex, mACC:mid anterior cingulate cortex, PFC: prefrontal cortex, aIC: anterior insular cortex, pINS : posterior insula, aINS : anterior insula, DLPFC: dorsolateral prefrontal cortex, pre SMA:pre supplementary motor area, SMA: supplementary motor area, IFJ: inferior frontal junction, DMPFC: dorsomedial prefrontal cortex, Crus I / II: cerebellar Crus I / Crus II, VIIb: cerebellar lobule VIIb, VIIIa / VIIIb: cerebellar lobule VIIIa / VIIIb, Vermal lobule V: cerebellar vermis lobule V, Lobules I–IV, VI, VII: cerebellar lobules I–IV, VI, VI,I VSI: Visceral Sensitivity Index, PCS: Pain Catastrophizing Scale, GI: gastrointestinal, IBS: irritable bowel syndrome, ReHo: Regional Homogeneity, ALFF: Amplitude of Low Frequency Fluctuations, FC: Functional Connectivity, VMHC: Voxel Mirrored Homotopic Connectivity, DMN: Default Mode Network, MPFC: Medial Prefrontal Cortex, PCC: Posterior Cingulate Cortex, IPC: Inferior Parietal Cortex , MFC: Medial Frontal Cortex, ACC: Anterior Cingulate Cortex, dACC: Dorsal Anterior Cingulate Cortex, vACC: Ventral Anterior Cingulate Cortex, ORBsup / ORBsupmed: Superior / Medial Orbitofrontal Cortex, IPL: Inferior Parietal Lobule, SAS: Self Rating Anxiety Scale, SDS: Self Rating Depression Scale, SN: salience network, SMN: sensorimotor network , vACC: ventral anterior cingulate cortex, DC: degree centrality, HC: healthy controls, DEP IBS: depressed IBS people, nDEP IBS: non depressed IBS people, IBS D: diarrhea predominant IBS HAMD: Hamilton Depression Rating Scale

**Table 3:** Brain regions with altered activity and connectivity in patients with ibs linked with depression, anxiety, and cognition, and their associated functional networks

Anatomical Location	Brain regions	Associated network	Network Function
medial prefrontal cortex	pACC	SN(Doucet et al., 2025; Fernandes et al., 2024; Magioncalda et al., 2015)	The SN helps identify important stimuli and regulates brain activity to prioritize attention and manage emotional responses.
Cingulate cortex	aMCC		
anterior part of the insular cortex	aINS		
lateral sulcus	insula		
frontal lobes	PFC	ECN(Causse et al., 2022; Sugaya et al., 2021)	The ECN involved in higher cognitive functions like decision-making, problem-solving, and goal-directed behaviour.
lateral part of the prefrontal cortex	DLPFC		
junction of the inferior frontal gyrus and the precentral gyrus in the frontal lobe	IFJ		
posterior part of the insular cortex	pINS	SMN(Doucet et al., 2025; Fernandes et al., 2024; Wei & Bao, 2013) Brainstem regions interacting with sensorimotor networks(Singh et al., 2022)	The SMN involved in controlling voluntary movements and processing sensory information from the body.
medial part of the frontal lobe	SMA		
frontal lobe	precentral gyrus		
brainstem, between the forebrain and hindbrain	midbrain		
cerebellum	Crus II	Cognitive–affective cerebellar regions linked to DMN(Guell & Schmahmann, 2020) DMN(Chen et al., 2024; Doucet et al., 2025; Pan et al., 2022; Yan et al., 2009)	The DMN active during rest and self-referential thinking, involved in mind-wandering, memory consolidation, and internal thought processes. The DMN active during rest and self-referential thinking, involved in mind-wandering, memory consolidation, and internal thought processes.
cerebellum	Crus I		
cerebellum	Crus VII		
medial prefrontal cortex	DMPFC		
medial part of the orbitofrontal cortex in the frontal lobe	ORBsupmed		
medial parietal lobe	precuneus		
lower part of the anterior cingulate cortex	vACC		
medial temporal lobe	hippocampus		
superior part of the frontal lobe	medial superior frontal gyrus		
parietal lobe	inferior parietal lobule		

Pregenual anterior cingulate cortex (*pACC*), anterior midcingulate cortex (*aMCC*), Prefrontal cortex (*PFC*), Dorsolateral Prefrontal Cortex (*DLPFC*), Posterior Insula (*pINS*), Supplementary Motor Area (*SMA*), anterior insula (*aINS*), Inferior Frontal Junction (*IFJ*), Dorsomedial Prefrontal Cortex (*DMPFC*), Medial Superior Orbital Gyrus (*ORBsupmed*), Ventral Anterior Cingulate Cortex (*vACC*)

### Task-Based fMRI results

#### Anxiety and pain

Compared with healthy controls, in IBS patients, anxiety was associated with pain-related activation in the right anterior midcingulate cortex (aMCC) and pregenual anterior cingulate cortex (pACC), with greater activation in these regions correlating with higher anxiety scores. Additionally, anxiety was linked to activation in the right cerebellar Crus II region in response to non-painful stimuli, with increased cerebellar activation also corresponding to higher levels of anxiety (Elsenbruch et al., 2010; Rosenberger et al., 2013).

#### Depression and pain

In IBS patients depression correlated with pain-stimulated activation in the left prefrontal cortex (PFC) and cerebellar regions, and non-painful distension activated the right cerebellum, specifically Crus II and lobule VIII, and vermal lobule V during painful rectal distension (Elsenbruch et al., 2010; Rosenberger et al., 2013). For cognitive tasks, IBS patients exhibit distinctive patterns of brain activation compared with healthy controls. During set-shifting tasks, there is increased activation in the right pre-supplementary motor area (SMA) and left dorsolateral prefrontal cortex (DLPFC), alongside reduced right DLPFC activation during error feedback processing (Aizawa et al., 2012). Executive function tasks revealed deactivation in the right thalamus and increased activity in the right dorsomedial PFC. During altering tasks, IBS patients demonstrated greater activity in the left anterior midcingulate cortex (LaMCC), bilateral anterior insula (aINS), and right posterior insula (pINS), but deactivation in the left precentral gyrus and reduced activity in the left superior frontal gyrus during orienting tasks(Hubbard et al., 2015).

### Resting-State fMRI results

#### Anxiety and Depression

Depression in IBS patients (DEP-IBS) was linked to reduced activity in the left insula and increased activity in the left precentral gyrus, with reduced insula activity correlating with greater depression severity. DEP-IBS patients also show altered connectivity patterns, including stronger insula connections with regions such as the left inferior parietal lobule and right inferior occipital gyrus but weaker connections with the postcentral gyrus and supplementary motor area. These altered connectivity patterns were also linked to higher anxiety and depression scores (Li et al., 2021). Anxiety-depression symptoms correlated with increased ALFF in the right midbrain and decreased ALFF in limbic structures like the anterior cingulate cortex (ACC) insula, and thalamus (Chen et al., 2021).

Adjusting for anxiety and depression abolishes differences in DMN functional connectivity in IBS patients compared to healthy controls. For example, reduced interhemispheric connectivity in the ventral ACC (vACC) and ALFF differences in both the dorsal and ventral ACC become non-significant when accounting for mood disorders (Rongfeng Qi, Chang Liu, Jun Ke et al., 2016; Rongfeng Qi, Chang Liu, Yifei Weng et al., 2016). However, other studies have indicated that when depression and anxiety are accounted for as covariates, no significant differences in resting-state functional connectivity (RSFC) were found between IBS patients and controls (Icenhour et al. 2017; Ke et al. 2015; Qi et al. [Bibr CR86][Bibr CR56], c).

## Discussion

Our review demonstrates that changes in brain networks, especially those involved in emotional regulation and executive function, are closely linked to anxiety, depression, and cognitive difficulties in patients with IBS compared to healthy controls. This is in line with previous research, which has also suggested that IBS is associated with altered brain connectivity, particularly in networks related to pain perception and emotional processing(Nisticò et al., 2022). However, it should be noted that in the studies we reviewed cognitive tasks such as set-shifting, error feedback processing, and attentional orienting, mainly assess executive and attentional control processes in situations involving pain anticipation, salience processing, and awareness of internal bodily signals. Therefore, cognitive changes identified in IBS thus far are largely related to emotional and visceral processing, rather than reflecting alterations in basic cognitive functioning. Future studies could clarify whether IBS-related cognitive effects extend beyond emotional and visceral domains (Nisticò et al., 2022).

One key finding from our review was increased activity in the salience network (SN) in IBS, especially the pACC and anterior insula. These regions play a critical role in detecting and integrating internal bodily sensations (such as visceral pain) and allocating attention to salient stimuli (Palomero-Gallagher et al., 2019; Zhang et al., 2024). Alterations in the SN could be a factor in the hyperawareness of gastrointestinal sensations commonly reported in IBS, contributing to increased symptom perception and anxiety (Elsenbruch et al., 2010). Furthermore, We found activity in the right DLPFC is linked to anticipating pain, which suggests it plays a role in controlling pain signals (Aizawa et al., 2012; Lorenz et al., 2003). The DLPFC is known for its role in top-down modulation of pain and emotion, and reduced activation during error feedback processing may suggest impaired cognitive flexibility in IBS patients, which could contribute to rumination and difficulty adapting to changing internal states (Qiao et al., 2024).

Disruption in the default mode network (DMN), especially reduced connectivity between the precuneus and anterior cingulate cortex (ACC), can be associated with impaired self-focused information processing and emotional control (Rongfeng Qi, Jun Ke et al., 2016). The precuneus is involved in self-awareness and internally directed thought, while the ACC integrates emotional and cognitive information (Dadario & Sughrue, 2023; Stevens et al., 2011). Disconnection between these regions may contribute to the heightened emotional distress seen in IBS.

Changes in the sensorimotor network (SMN), including regions such as the precentral gyrus and thalamus, suggest altered processing of sensory information and motor responses in individuals with IBS (Li et al., 2021). The SMN plays a key role in integrating somatic and visceral sensory inputs with motor output (Wasaka & Kakigi, 2019). Dysregulation in this network may contribute to visceral hypersensitivity and motor dysfunction, such as bowel irregularities, commonly reported in IBS (Barbara et al., 2011). We also found differences in brain connectivity during both task and resting-state based activities. IBS patients showed reduced connectivity in the DMN, especially between the precuneus and ACC, which are important for emotional regulation and self-awareness (Rongfeng Qi, Jun Ke et al., 2016; Yang et al., 2023). Changes in the SMN and SN suggest that IBS affects both visceral processing and cognitive control (Zhao et al., 2023).

Finally, the fronto-limbic circuits seem to play a key role in linking pain and emotions. Reduced connectivity in areas like the hippocampus, ACC, and SN may make it difficult for the brain to regulate pain and process emotions, which can lead to symptoms like depression and anxiety(Qi et al. 2016a, b, c, d; Zhang et al. 2018).

It is important to note that almost all of the reviewed studies were cross-sectional, so the links between IBS-related symptoms and changes in brain connectivity are associations and not proven cause-and-effect relationships with clear directionality. For instance, personality traits placing participants at higher risk for IBS could be confounded by mood and cognitive changes (Muscatello et al., 2016). Alternatively, chronic IBS symptoms could gradually change brain networks over time, predisposing participants to certain mood and cognitive impairments. Finally, anxiety and depression may directly modulate the processing of bodily sensations and pain, such that IBS is a symptom of depression (Elsenbruch, 2011). In line with this view, several studies report that group differences in functional connectivity between IBS patients and healthy controls are reduced or abolished when controlling for anxiety and depression (Rongfeng Qi, Chang Liu, Jun Ke et al., 2016; Rongfeng Qi, Chang Liu, Yifei Weng et al., 2016). This suggests that mood symptoms may do more than confound the results; they could serve as key mediators of change in brain networks in IBS, though it also raises the possibility that there may be no clearly identifiable IBS-specific connectivity signature separate from the well-established patterns associated with mood disorders.

Functional MRI studies further show that anxiety and depression influence neural responses to visceral pain, increasing activation in emotion- and salience-related regions, with IBS patients showing altered emotional modulation compared with controls (Elsenbruch et al., 2010; Icenhour et al., 2017; Nisticò et al., 2022). Contemporary reviews emphasize the strong bidirectional relationship between common mental disorders and IBS, including their shared effects on central network dynamics (Fadgyas-Stanculete et al., 2014). These observations support a mediational framework in which affective symptoms mediate the relationship between IBS symptoms and brain network alterations. In this model, interventions targeting mood such as cognitive‑behavioral therapy (CBT) or mindfulness‑based stress reduction (MBSR) may have downstream effects on neural network organization, improving both emotional regulation and gastrointestinal symptoms(Jacobs et al., 2021; Oraki et al., 2020). For example, CBT responders show reduced functional connectivity in sensorimotor, brainstem, salience, and default mode networks, and these changes may be linked to shifts in the gut microbiome(Jacobs et al., 2021). Treating mood as a mediator rather than a mere confounder highlights the value of integrated approaches that address both emotional and visceral aspects of IBS. CBT is evidence‑based for IBS and recommended within biopsychosocial care models, and recent clinical frameworks emphasize that managing co-occurring anxiety and depression is essential for optimizing IBS outcomes(Staudacher et al., 2023).

However, it remains uncertain whether IBS pathophysiology causes connectivity changes or if these changes result from adaptive processes, behavioral alterations, or medication effects. Current evidence, derived largely from cross-sectional designs, cannot definitively distinguish whether observed network changes are specific to IBS pathophysiology or primarily reflect the neural correlates of comorbid anxiety and depression. Future research should aim to clarify these effects using larger samples, longitudinal studies, and analytic approaches capable of separating mood- and IBS-specific neural contributions. Such studies would clarify whether IBS exhibits unique brain network alterations or if observed changes largely reflect the impact of affective comorbidity.

### Influence of analytical approach on reported findings

An important factor in neuroimaging research is how imaging and clinical data are analysed, as methodological choices can systematically influence the level and type of neural effects detected. Some studies integrate imaging and clinical data within a single analytical framework, allowing a more comprehensive characterisation of brain–symptom relationships (Aizawa et al., 2012). This approach facilitates clearer mapping between brain activity and clinical symptoms. In contrast, analysing imaging and clinical data separately may allow focused examination of specific effects but can limit insight into their interdependence, potentially contributing to variability in reported findings across studies.

When studies are considered by analytical approach, clear patterns emerge. Task-based and ROI- or seed-based studies, which are typically hypothesis-driven, predominantly focus on specific affective–visceral regions, including the anterior cingulate cortex, insula, amygdala, thalamus, and prefrontal cortex (Aizawa et al., 2012). These studies commonly include clinical or behavioural measures as covariates and tend to report connectivity alterations linked to pain processing, emotional regulation, and mood-related symptoms. This suggests that ROI-driven approaches are particularly sensitive to localised, symptom-related neural reactivity in IBS.

In contrast, resting-state studies using whole-brain analytical methods—including independent component analysis, graph theory, network-based connectivity, voxel-mirrored homotopic connectivity (VMHC), regional homogeneity (ReHo), and amplitude of low-frequency fluctuations (ALFF)—more frequently identify alterations at the network level (Li et al., 2021). These approaches highlight disruptions within large-scale systems such as the default mode, salience, and sensorimotor networks, as well as broader properties of network organisation, global integration, and interhemispheric connectivity. Rather than contradicting ROI-based findings, these results point to complementary effects, with whole-brain analyses capturing more distributed patterns of dysregulation.

Taken together, these methodological differences help explain variability and apparent inconsistencies across the IBS neuroimaging literature. ROI-based approaches appear more sensitive to localised, symptom-linked neural responses, whereas whole-brain analyses capture distributed network-level alterations. While these approaches are complementary when combined, heterogeneity in analytical strategies and reporting limits the ability to draw comprehensive conclusions across studies. Mapping regional findings onto large-scale brain networks suggests that IBS-related neural alterations involve both circuit-specific affective–visceral regions and broader network-level disruptions. Rather than being redundant, these analytic approaches capture distinct neurobiological levels of the affective–visceral system, underscoring the need for greater methodological standardisation, clearer alignment between analytical strategy and research questions, and careful interpretation of findings within each study’s methodological framework.

### Limitations and future research directions

A limitation of this systematic review is the relatively small number of studies available, which reduces statistical power and the ability to detect robust effects. Additionally, several studies reported sex imbalances, with more female participants. However, reviewed studies did not examine or report sex differences in brain connectivity. Sex differences in brain connectivity have been reported in both healthy populations and IBS patients(Labus et al., 2013). Males showed increased connectivity among anterior cingulate, amygdala, and insula regions, whereas females showed increased connectivity to and from prefrontal modulatory areas, including medial and dorsolateral prefrontal cortex. These differences highlight that sex-related variation in salience, affective, and cognitive control networks may influence symptom–brain associations (Labus et al., 2013). A key limitation of the current literature is the lack of Functional MRI studies examining domain-general cognitive processes in IBS. Future research should expand cognitive batteries to include a broader range of cognitive paradigms that assess executive function, memory, and processing speed to provide a more comprehensive picture of the cognitive impact of IBS (Nisticò et al., 2022). Given the critical interface between mood, cognition and IBS symptoms, another limitation is the lack of studies that contemporaneously assess these domains in either task-based or resting state fMRI modalities. Better understanding of how these features are interrelated over time would be of great benefit to understand mechanisms and develop targeted interventions. Furthermore, a significant gap is the lack of reporting on dietary factors in the studies reviewed. Diet, which can impact both brain activity and IBS symptoms, was not accounted for, creating uncertainty about the role it may play in the observed results. Future work should aim to integrate multimodal fMRI approaches to simultaneously examine mood, cognition, and IBS symptoms, addressing this gap in the literature.

### Implications for Intervention

These findings show a bidirectional relationship between mood and cognitive dysfunction in IBS, with emotional dysregulation impacting cognition, and difficulties in cognition in turn making emotions harder to manage in the presence of IBS symptoms. The interplay between the DMN, SN, and ECN connectivity suggests that interventions targeting these interconnected brain regions could be helpful. For instance, CBT helps patients identify and modify unhelpful thoughts about their symptoms, which can reduce anxiety and excessive attention to bodily sensations. This may lower heightened activity in brain systems involved in salience and interoceptive processing(Ghaffari, 2025; Reynolds et al., 2023). CBT also supports cognitive control and coping by improving top-down regulation of emotional and self-focused processing, and it includes relaxation and stress-management strategies that can reduce stress-related gut sensitivity (Lackner et al., 2018). Although direct neuroimaging evidence for network-specific changes in IBS is still limited, clinical trials consistently show that CBT improves pain coping, reduces symptom severity, and enhances quality of life(Kinsinger, 2017; van der Schaaf et al., 2024). Alternatively, mindfulness-based programs, including Mindfulness-Based Cognitive Therapy (MBCT), may help patients become more aware of bodily sensations while reacting less emotionally. In IBS, this may reduce visceral hypersensitivity and unhelpful thought patterns, potentially improving regulation between the salience and default mode networks(Garland et al., 2012; Zernicke et al., 2013). Additionally, Transcranial Magnetic Stimulation (TMS) may be an effective non-pharmacological intervention for IBS. Existing TMS trials in IBS and other functional gastrointestinal disorders primarily target the dorsolateral prefrontal cortex (DLPFC), a key hub of the executive control network (ECN) (Algladi et al., 2015; G. Li et al. 2023a, b). Stimulation of the DLPFC can modulate ECN activity and exert downstream effects on pain-processing regions, including the anterior cingulate cortex and insula, which are core nodes of the SN (Algladi et al., 2015). By enhancing top down- cognitive control, prefrontal TMS may reduce visceral hypersensitivity and improve pain modulation in IBS patients (G. Li et al. 2023a, b).

## Conclusion

This review brings together evidence on mood symptoms, cognitive processing, and brain network alterations in IBS. By integrating these domains, we show that IBS is best understood as a condition involving dysregulation across multiple interacting brain networks, rather than isolated changes in a single system. Key networks affected include the default mode, salience, sensorimotor, and executive control networks.

Across studies, changes in these networks were closely linked to emotional regulation, visceral pain processing, and context-dependent executive and attentional control. Evidence for broad, domain-general cognitive impairment was limited. Importantly, several studies reported that differences in brain connectivity between IBS patients and healthy controls were reduced or no longer present after accounting for anxiety and depression. This suggests that mood symptoms may play a central role in shaping brain network alterations in IBS, and that IBS and affective symptoms may partly reflect shared underlying neural pathways, particularly within affective–visceral and salience-related systems.

Together, these findings support a framework in which anxiety and depression mediate the relationship between IBS symptoms and altered brain connectivity. This perspective highlights the close interaction between emotional state, cognitive control, and visceral processing. Future research should aim to separate neural signatures related to mood and cognition within IBS using larger samples and longitudinal designs.

## Supplementary Information

Below is the link to the electronic supplementary material.

Supplementary Material 1 (DOCX 19.9 KB)

## Data Availability

No datasets were generated or analysed during the current study.
