# Supplementary material for: A systematic review of associations between functional connectivity, mood and cognition in patients with irritable bowel syndrome
Source: Brain Imaging Behav. 2026 Apr 7;20(2):71. doi: 10.1007/s11682-026-01135-9 (PMC13056754; doi:10.1007/s11682-026-01135-9)
Supplement: Supplementary file 1 — Supplementary Material 1 (DOCX 19.9 KB) [file 11682_2026_1135_MOESM2_ESM.docx]

**SupplementaryTable 1: Risk of Bias**

| Author/year | Research question clarity | Recruitment methods | Eligibility criteria | Participants characteristics | Imaging technique | Control group | Risk of bias |
| --- | --- | --- | --- | --- | --- | --- | --- |
| Elsenbruch, S,2010 | * | * | * | * | * | * | Low |
| Aizawa, E,2012 | * | * | * | * | * | * | Low |
| Rosenberger, C, 2013 | * | * | * | * | * | * | Low |
| Hubbard, C,2015 | * | * | * | * | * | * | Low |
| Ke,J, 2015 | * | * | * | * | * | * | Low |
| Qi,R, 2016a | * | * | * | * | * | * | Low |
| Qi,R, 2016b | * | * | * | * | * | * | Low |
| Qi, R,2016c | * | * | * | * | * | * | Low |
| Qi, R,2016d | * | * | * | * | * | * | Low |
| Icenhour, A,2017 | * | * | * | * | * | * | Low |
| Li,J, 2021 | * | * | * | * | * | * | Low |
| Chen,X, 2021 | * | * | * | * | * | * | Low |
